# The impact of CPR and AED training on healthcare professionals' self-perceived attitudes to performing resuscitation

**DOI:** 10.1186/1757-7241-20-26

**Published:** 2012-04-05

**Authors:** Marie-Louise Södersved Källestedt, Anders Berglund, Johan Herlitz, Jerzy Leppert, Mats Enlund

**Affiliations:** 1 Uppsala University, Centre for Clinical Research, Västerås, Sweden; 2 Department of Medical Epidemiology and Biostatistics, Karolinska Institutet, Stockholm, Sweden; 3 Centre for Pre-hospital research, Western Sweden University College of Borås and Sahlgrenska University Hospital, Gothenburg, Sweden; 4 Centre for Clinical Research, Central Hospital, Västerås,, S-721 89 Västerås, Sweden

**Keywords:** Education, Cardiopulmonary resuscitation, Attitude, Defibrillators, Health personnel

## Abstract

**Background:**

Healthcare professionals have shown concern about performing mouth-to-mouth ventilation due to the risks to themselves with the procedure. However, little is known about healthcare professionals' fears and attitudes to start CPR and the impact of training.

**Objective:**

To examine whether there were any changes in the attitudes among healthcare professionals to performing CPR from before to after training.

**Methods:**

Healthcare professionals from two Swedish hospitals were asked to answer a questionnaire before and after training. The questions were relating to physical and mental discomfort and attitudes to CPR. Statistical analysis used was generalized McNemar's test.

**Results:**

Overall, there was significant improvement in 10 of 11 items, reflecting various aspects of attitudes to CPR.

All groups of health care professionals (physicians, nurses, assistant nurses, and "others" = physiotherapists, occupational therapists, social welfare officers, psychologists, biomedical analysts) felt more secure in CPR knowledge after education. In other aspects, such as anxiety prior to a possible cardiac arrest, only nurses and assistant nurses improved.

The concern about being infected, when performing mouth to mouth ventilation, was reduced with the most marked reduction in physicians (75%; *P *< 0.001).

**Conclusion:**

In this hospital-based setting, we found a positive outcome of education and training in CPR concerning healthcare professionals' attitudes to perform CPR. They felt more secure in their knowledge of cardiopulmonary resuscitation. In some aspects of attitudes to resuscitation nurses and assistant nurses appeared to be the groups that were most markedly influenced. The concern of being infected by a disease was low.

## Introduction

Cardiac arrest may occur anywhere in a hospital, and be discovered by any healthcare professional [1]. Although being difficult to prove, it is not unlikely that the attitude towards cardiopulmonary resuscitation (CPR) among health care professionals is of importance for the chance of survival after cardiac arrest. Some of these professionals may be concerned about of the potential risks to themselves of starting CPR. To our knowledge, there has been no previous study on the effects of CPR training and education to attitudes in cardiopulmonary resuscitation, covering the spectrum of all healthcare professionals. Thus, the purpose of this study was to investigate the impact of CPR training upon health care professionals' attitudes to performing resuscitation. A secondary aim was to describe the eventual changes in attitudes to CPR among various groups of healthcare professionals.

## Methods

The study was approved by the Uppsala Regional Ethics Committee, Sweden (2006/201).

### Study participants

Healthcare professionals were recruited from two hospitals (one minor and one medium-sized hospital) in the county of Västmanland, central in Sweden. Only individuals actively working at the time were eligible for inclusion in the study (i.e. those on maternity or sick leave were not included). A total of 2614 out of 3165 (83%) employed healthcare professionals agreed to participate in the education programme, of these, 82% completed the follow-up afterwards. To be eligible for the follow-up test, there was a requirement to have actually participated in CPR training. All healthcare professionals had to take part in CPR training at some time during the year. The questionnaire contained one particular question of vital importance: "Have you performed cardiopulmonary resuscitation on an adult or a child?" If the healthcare professional answered yes to either of the two alternatives, they were requested to continue answering questions about their attitude and their worry to be infected when performing mouth to mouth ventilation. All healthcare professionals were also asked: "Are you certain that you know how to use an automated external defibrillator"?

**Figure 1:**
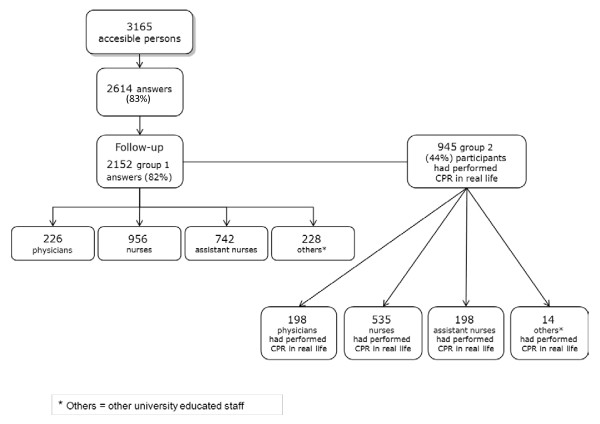
**Flowchart of participants in the study**.

The study population consisted of two groups: Group 1 taking part in the CPR training (n = 2152), group 2 taking part in the training and had performed CPR in real life (n = 945) (Figure 1). These two groups were then divided into four subgroups, as follows:

1. Physicians

2. Nurses (including midwives),

3. Other university-educated staff (including physiotherapists, occupational therapists, social welfare officers, psychologists and biomedical analysts)

4. Assistant nurses (including keepers),

### The training programme

The cardiopulmonary resuscitation training presented to the participants followed the guidelines from the Swedish Resuscitation Council. In Sweden, the use of AEDs is taught in a 4-hours course that also includes theory and practical training in basic CPR, use of oxygen and ventilation with mouth-to-mask technique, and use of suction devices for clearing of the airways [2].

### Questionnaire

A questionnaire was designed to collect information about healthcare professionals' attitudes to and experiences of performing CPR (cf.: Appendix). To ensure validity and clarity, twenty-two healthcare professionals from the two participating hospitals were invited to complete the questionnaire. They were then requested to reflect over any words in the questions that they did not understand, or sentences that could be understood in different ways. After synthesis of these reflections five individual interviews were conducted, (by the first author), to guarantee that participants understood the questions in the same way [3]. This resulted in some amendments to the wording in the questionnaire. The questions were then overviewed by a person with knowledge in CPR and by one person with knowledge in pedagogy and attitudes. The revised questionnaire was then sent to all healthcare professionals at the two hospitals. A covering letter was enclosed to explain the nature of the questionnaire, and that participants would be asked to answer the same questions again approximately two years later, depending on when each individual received their CPR training. The usual delay for answering the questionnaire was one to three months from participating in CPR training. All the participants returned the completed questionnaires via internal mail in paper format directly to the investigator.

The questions in Appendix had three options; yes, no, do not know.

### Statistics

The attitude changes were based on information provided before and after training with individually matched data. In matched-pairs data, the McNemar's test can be applied only when the outcome has two possible categories. However, since the outcome in this study was three (yes, no, do not know) or more for all variables, and not ordinal, the generalized McNemar's test for homogeneity of the marginal distributions was used [4]. In subsequent analyses, the attitude changes were analyzed stratified by health occupation groups. The *P*-values were two-sided, and an alpha level of 0.05 was considered to be statistically significant. All analyses were performed using R 9.2 [5].

## Results

The age of the participants were in median 40 years (range 20-68), and they had a working experience of 18 years (range a few months to 46 years). There were no statistical associations between the healthcare professionals' attitudes and their ages and work experiences. A total of 945 (44%) of the participants had performed CPR. Of these, 903 participants had performed CPR on adults, and 247 on children (which means that some of the healthcare professionals had performed both).

Over all, there were improvements in ten out of eleven aspects of attitudes. Training significantly influenced attitudes among the nurses and assistant nurses. Assistant nurses increased their secure attitude in CPR knowledge by 19 percentage points (from 49 up to 68%, *P *< 0.001), and they reported that they knew what to do if a cardiac arrest would occur. The physicians' attitudes of feeling secure in their CPR knowledge were at the same level as for the nurses (70% felt secure after education vs. 52% before, Table 1). The training affected the nurses' and assistant nurses' attitudes, so that a majority had a feeling of knowing what to do if a cardiac arrest would occur (Table 1). All healthcare professionals increased their positive answers from 24 to 67% (*P < 0.01*) regarding knowing how to use an AED after CPR training.

**Table 1:** Differences in attitudes after CPR education by all healthcare professions (Group 1, taking part in CPR training)

Question ^1^	Physicians (n = 226)			Nurses (n = 956)			Assistant nurses (n = 742)			Others ^2^(n = 228)			Total (n = 2152)		
	**%**	**Diff**.	***P***	**%**	**Diff**.	***P***	**%**	**Diff**.	***P***	%	Diff.	*P*	%	Diff.	*P*
	
Sure how to react	74	+10	0.03	67	+9	< 0.001	60	+13	< 0.001	30	+5	NS	60	+10	< 0.001

Not nervous	53	-5	NS	54	+3	0.01	53	+8	< 0.001	21	+2	NS	49	+4	< 0.001

Duty to intervene	98	+3	NS	97	-2	NS	96	+3	NS	92	+4	NS	96	+0	NS

Secure in CPR knowledge	70	+18	< 0.001	71	+14	< 0.001	68	+19	< 0.001	31	+7	0.04	65	+16	< 0.001

Not anxious	69	+0	NS	65	+10	< 0.001	64	+13	< 0.001	29	0.0	NS	60	+9	< 0.001

Know what to do if cardiac arrests occur	90	+5	NS	90	+7	< 0.001	88	+12	< 0.001	67	+5	NS	86	+8	< 0.001

Act instinctively	73	+10	NS	76	+5	0.02	72	+5	NS	49	+5	NS	71	+5	< 0.001

Chance to help	87	+4	NS	91	+3	NS	92	+5	0.006	72	+9	NS	88	+4	< 0.001

Need to have things under control	62	+0	NS	67	+4	NS	51	+1	NS	49	+9	0.04	58	+3	< 0.001

Manage to take control of the situation	89	+4	NS	68	+8	< 0.001	43	+14	< 0.001	38	+7	NS	57	+10	< 0.001

Important to use gloves	43	+8	NS	32	-0	NS	27	+5	0.008	9	-0.0	NS	28	+2	< 0.001

^1 ^See the appendix for the complete questions

^2 ^Other university educated staff, including physiotherapists, occupational therapists, social welfare officers, psychologists and biomedical analysts

% Proportion of participants with the respective attitude after education

Diff. Change in proportion of participants before and after education in CPR

*NS *Not Significant

The physicians' attitudes were less influenced by education than the other healthcare professionals. In the group of healthcare professionals that had performed CPR in real life the physicians reduced their fear for infection transmission during CPR from 90% to 14% (*P *< 0.001) (fear of being infected when performing mouth-to-mouth ventilation). Among nurses, 82% had a fear of being infected before and 17% after training in CPR.

With training, the overall proportion of healthcare professionals having lack of anxiety, if they thought that they might need to perform CPR when arriving at work, increased from 51 to 60% (*P *< 0.001). But when looking at specific professions, only nurses and assistant nurses reduced their anxiety. The group of "other" healthcare professionals did not change in anxiety after training. This group also had the least who were not anxious (21%).

## Discussion

This study evaluated the influence of education on the attitudes to resuscitation across the whole spectrum of different health care professions within two hospitals with wide representation. The major message was that we found improvement in ten out of eleven items, reflecting various aspects of attitudes to cardiopulmonary resuscitation. To the best of our knowledge no similar large scale effort with intention to measure attitudes to resuscitation has ever been performed.

In some aspects it appeared as all groups of health care providers improved. Such an aspect was "Feeling secure in CPR knowledge". However, in other aspects such as "Feeling anxious about an eventual resuscitation event on their way to work" nurses and assistant nurses improved significantly, whereas physicians (low degree of anxiety) and "other health care professionals" (high degree of anxiety) did not change in their attitudes. In total 96% felt a duty to intervene already on beforehand (the only item that did not change).

## General discussion

We found it worth studying the attitudes to cardiopulmonary resuscitation of all different healthcare professionals in the two hospitals, as all groups are close to patients. They should, therefore, be able to initiate CPR and also to use AED. Accordingly, hospital leaders have an obligation to ensure that all their staff receives training in resuscitation [6].

Interestingly, nurses and assistant nurses tended to change their attitudes most among all healthcare professions. This may positively affect their willingness to initiate CPR in an actual resuscitation situation. One question in the questionnaire required the participants to estimate themselves; "I know what to do if cardiac arrests occur". We expected that over 80% of the group of "other healthcare professionals" would also have a more positive attitude after CPR training to knowing what to do if a cardiac arrest occurs. However, despite the training they received, only two-thirds of the group of other university-educated staff would know what to do in an actual CPR situation. It remains to be investigated whether concerns may be decreased with more simulation training during the annual training intervention. The physicians remained stable in their attitudes, and they only increased their feeling of security in their CPR knowledge.

Physicians, nurses and assistant nurses increased their confidence in being certain how to react in a CPR situation, up to 60-74% after CPR training. Nonetheless, we expected a higher proportion to feel secure after training. Consequently, there is room for innovation in the educational programmes. One way may be to adapt CPR education to the profession. One might, though, argue that these increased efforts may provide a better "pay off" to the groups of professionals who are closest to the patient.

In Hong Kong, there have been two deaths among personnel who performed resuscitation on a patient known to be suffering from severe acute respiratory syndrome. The risk of becoming infected have influenced medical students in Hong Kong [7], whereas in a study on laypeople 93% were not afraid of initiating CPR, even though there was a possibility that the person with cardiac arrest had AIDS [8]. Other potential risks for infection transmission during CPR can be HIV [9], staphylococcus aureus [10], and herpes simplex infections [11]. Objectively, there is a minimal risk that healthcare professional may become infected by HIV or hepatitis when performing mouth-to-mouth ventilation [12]. In our study physicians' and nurses' fears of being infected while performing mouth-to-mouth ventilation were significantly reduced after education/training in cardiopulmonary resuscitation. This agrees to Bhanji et al. who suggested that the willingness to perform CPR can be overcome with education [13]. It remains to be seen if the attitudes expressed in this study can be applied into real-life situations. Hopefully, the changed attitudes will increase the chance of a cardiac arrest patient to receive optimal resuscitation.

Future studies concerning the attitudes among physiotherapists, occupational therapists, social welfare officers, psychologists and biomedical analysts, and their needs of tailor made CPR training are warranted. Such studies must, however, take into consideration that resources are limited, and must be weighed against the fact that other groups of professionals, closer to patients, also have the scope to increase their knowledge, skills and attitudes.

### Strengths and limitations

A strength is the population based prospective study design with a large number of participants. It reports subjective data, relying merely on the view of the rescuer, which should be kept in mind when evaluating our findings. We have previously presented relatively good outcome in theoretical CPR knowledge from training, which is of some interest when interpreting the current results [14]. However, no objective analysis of the number of infections among the healthcare professionals was undertaken, which is a limitation. Nevertheless, we believe that our study presents new data regarding healthcare professionals' attitudes to perform CPR, which may be generalised to at least the current situation in Sweden if not the entire Scandinavia or western world.

## Conclusion

Taken together, in this hospital-based setting, we found a positive outcome of education and training in CPR concerning healthcare professionals' attitudes to perform CPR and to use AEDs. They felt more secure in their knowledge of cardiopulmonary resuscitation. In some aspects of attitudes to resuscitation nurses and assistant nurses appeared to be the groups that were most markedly influenced. The concern of being infected by a disease was low.

## Competing interests

The authors declare that they have no competing interests, financial or otherwise, in the publishing of this manuscript.

## Authors' contributions

MLSK participated in the design and planning of the study, collected the data, participated in the statistical analysis, wrote the manuscript draft, and co-ordinated the subsequent versions of the manuscript. AB performed the statistical analysis and revised the manuscript. JH revised the study manuscript and made important additions. JL participated in the design and planning of the study. ME participated in the design and planning of the study and was involved in drafting the manuscript and the statistical analysis. All authors read and approved the final manuscript.

## Appendix

Imagine that you are on your way to work and you know that you will have some patients with cardiac disease at your work place. There is a substantial risk that some of these patients will suffer a cardiac arrest and that you will have to perform cardiopulmonary resuscitation, what do you feel faced with this situation?

Questions in Table 1:

Question: I would feel unsure of how to react

Question: I would feel nervous to be brought face to face with the situation

Question: I would consider it my duty to intervene if necessary

Question: I would feel secure in my cardiopulmonary resuscitation knowledge

Question: I would feel anxious

Question: I know what to do if cardiac arrests occur

Question: I would act instinctively

Question: I would see it as a chance to help

Question: I would have a need to have things under control

Question: If necessary I would manage to take command of the situation

Question: It is important that I use gloves

Reply alternatives: yes, no, do not know.
